# Scientometric and patentometric analyses to determine the knowledge landscape in innovative technologies: The case of 3D bioprinting

**DOI:** 10.1371/journal.pone.0180375

**Published:** 2017-06-29

**Authors:** Marisela Rodríguez-Salvador, Rosa María Rio-Belver, Gaizka Garechana-Anacabe

**Affiliations:** 1 Tecnologico de Monterrey, Escuela de Ingeniería y Ciencias, Monterrey, Nuevo León, Mexico; 2 Foresight, Technology and Management (FTM) Group. Industrial Organization and Management Engineering Department, University College of Engineering of Vitoria-Gasteiz, University of the Basque Country UPV/EHU, Basque Country, Spain; 3 Foresight, Technology and Management (FTM) Group. Industrial Organization and Management Engineering Department, Escuela Universitaria de Estudios Empresariales de Bilbao, University of the Basque Country UPV/EHU, Basque Country, Spain; Institut Català de Paleoecologia Humana i Evolució Social (IPHES), SPAIN

## Abstract

This research proposes an innovative data model to determine the landscape of emerging technologies. It is based on a competitive technology intelligence methodology that incorporates the assessment of scientific publications and patent analysis production, and is further supported by experts’ feedback. It enables the definition of the growth rate of scientific and technological output in terms of the top countries, institutions and journals producing knowledge within the field as well as the identification of main areas of research and development by analyzing the International Patent Classification codes including keyword clusterization and co-occurrence of patent assignees and patent codes. This model was applied to the evolving domain of 3D bioprinting. Scientific documents from the Scopus and Web of Science databases, along with patents from 27 authorities and 140 countries, were retrieved. In total, 4782 scientific publications and 706 patents were identified from 2000 to mid-2016. The number of scientific documents published and patents in the last five years showed an annual average growth of 20% and 40%, respectively. Results indicate that the most prolific nations and institutions publishing on 3D bioprinting are the USA and China, including the Massachusetts Institute of Technology (USA), Nanyang Technological University (Singapore) and Tsinghua University (China), respectively. *Biomaterials* and *Biofabrication* are the predominant journals. The most prolific patenting countries are China and the USA; while Organovo Holdings Inc. (USA) and Tsinghua University (China) are the institutions leading. International Patent Classification codes reveal that most 3D bioprinting inventions intended for medical purposes apply porous or cellular materials or biologically active materials. Knowledge clusters and expert drivers indicate that there is a research focus on tissue engineering including the fabrication of organs, bioinks and new 3D bioprinting systems. Our model offers a guide to researchers to understand the knowledge production of pioneering technologies, in this case 3D bioprinting.

## Introduction

Understanding the scientific and technological dynamics behind innovative technologies is crucial to help organizations in their research and development (R&D) strategic planning. It is highly valuable, both for present and future decision making, to determine the knowledge landscape of a research field. This includes statistics relating to the growth of the field—such as the countries leading in development, the institutions that are the most prolific and the prominent journals—which can be used to determine global technology trends. Over the years, many studies have assessed the qualitative evolution of scientific technology research and patents [1–2]. However, this research differs from others [2–3], as it is integrating a novel competitive technology intelligence (CTI) cycle, which focuses on the analysis of both scientific and technological output and is enriched by the incorporation of experts’ perspectives at different stages of a project.

Competitive intelligence is based on the systematic and ethical process of gathering, analyzing and transforming information into actionable knowledge in the context of the competitive environment of an organization. Its main aim is to support decision making and strategic planning [4]. When technological events are the main focus of analysis, different terms are used, such as CTI and technology intelligence [5]. Broadly, these terms refer to the process of monitoring the competitive and technological environment of an organization to support decisions related to market, innovation, design, and product development [6]. Knowledge produced by CTI constitutes an important “early warning” for research, development, and even for innovation [7]. In fact, this process is frequently considered to be a foundational task for any creative and innovative process [8]. The formal and systematic application of CTI has gained prominence in technology-based organizations where its influence on producing competitive advantages has been strongly evidenced, through everything from the anticipation of potential threats to the identification of relevant opportunities for innovation [9]. Feedback from experts can complement and enhance CTI process from the early stages, which include the search strategy definition through the analysis and final validation. In this context, a hybrid CTI + expert perspective data model is proposed in this research in order to perform an analysis of emerging technologies such as 3D bioprinting, a highly innovative technology.

Three-dimensional (3D) printing—also known as additive manufacturing, rapid prototyping or solid free-form technology—is a revolutionary technology that is bringing important changes to the world. It involves a process whereby objects are produced by fusing or depositing materials such as plastic, metal, ceramic, powder, liquid or even living cells, layer-by-layer from a digital file [10]. The American Society for Testing and Materials (ASTM International) defines it as “the process of joining materials to make objects from 3D model data, usually layer upon layer, as opposed to subtractive manufacturing methods” [11]. High quality products with complex geometries and minimal waste can be built, benefiting a variety of sectors [12]. Applications for 3D printing are found in many fields including education, aerospace, defense, architecture, transportation, the production of consumer products and healthcare [13].

Although 3D printing has been used for decades in prototyping [14], 3D bioprinting—which can be defined as a technique used to print living cells in a predesigned pattern [15]—is an innovative technology that is still in a nascent stage. The first patent in the field of 3D bioprinting was granted in 2006 to Clemson University; it protected an invention entitled “ink-jet printing of viable cells” that consists of a method for developing a viable cell matrix by ink jet printing onto a substrate [16]. It was not until 2014 that a commercialization of this technology was developed by the company Organovo Holdings, Inc. [17]. In that year, Organovo successfully launched the exVive3D Liver, a liver tissue model for medical and drug research [18].

3D bioprinting enables the production of elements that repair, replace or control functions within or on the human body, while 3D printing (the general technology without biocomponents) applied to the health sector serves to create prototypes, models, prostheses, pre-surgery planning tools, alignment jigs and surgical cutting templates. Unquestionably, 3D bioprinting represents a breakthrough technology that can be used to address health problems, and its applications are growing rapidly [13]. Important efforts are being devoted to the production of a broad array of state-of-the-art applications such as skin substitutes for burn wounds [19], elements for urethral reconstruction [20], and components to be used in place of bones, ears, windpipes, jaw bones, cell cultures, stem cells, blood vessels, vascular networks, tissues and—in the future—organs [10]. The goals behind recent developments in 3D bioprinting include from the successful application of the technology in bio-clinical research testing to more importantly its use for the production of fully functioning organs for transplant [21]. In 2013, a total of 118,114 solid organs were reported to have been transplanted worldwide. This represents an increase of approximately 3% over 2012. However, the scarce availability of organs is evident, as this quantity represents less than 10% of global need [22]: this is a deficit that could be significantly aided by the application of 3D bioprinting in organ production.

There is still the major challenge of producing not only a superior quality, biocompatible product for the human body, but also creating live tissues that retain their biological functions. In fact, the retention of vascularization is one of the biggest problems 3D bioprinting faces, as it is difficult to mimic the natural blood vessel network that is critical for the long term viability of any 3D tissue [23].

3D printing has the potential to radically transform the health industry and will generate major economic and societal impacts [24]. Because of this, scientific output and patent activity are rapidly growing in 3D bioprinting. Many studies have focused on the fundamentals and challenges of 3D bioprinting. Ventola [10] presented a review of current and future applications of this technology, indicating their potential benefits. Yoo [25] analyzed 3D bioprinting techniques and proposed new potential patenting areas. Also, Sheehan and colleagues [26] described a number of bioprinting patents in order to determine general trends following a qualitative process. Given the major impact that 3D bioprinting may have on human health, an awareness of the scientific and technological knowledge production regarding this technology is critical. Nevertheless, studies addressing this are still lacking. Recently, Trappey and colleagues [27] developed an approach to explore biomedical 3D printing technology trends, based on an analysis of US patents from 1980 to August 2014. This study did not consider scientific output nor expert interviews. A study using CTI methodology and applying scientometric and patentometric analyses on 3D bioprinting has not been undertaken yet. Zhou and colleagues [28] developed a CTI methodology using scientific and patent production where participation of experts in the field helped to identify research emphases and trajectories. However, their research focused on nano drug delivery systems. To fill this gap that has been discussed, and to outline the 3D bioprinting global knowledge landscape, the research presented here proposes a hybrid data model based on a CTI cycle integrated with expert perspectives. It is applied to the current state of R&D in 3D bioprinting and considers both scientific literature and patent information from 2000 to mid-2016 from the major authorities worldwide.

The main aims of this study are 1) to provide a novel data model to determine the knowledge landscape of emerging technologies; 2) to apply this model in order to identify scientific and technology trends in 3D bioprinting; 3) to determine the most prolific countries, organizations and journals in this domain; and 4) to identify the major research foci.

## Methods

### Development of a hybrid data model

**Fig 1:**
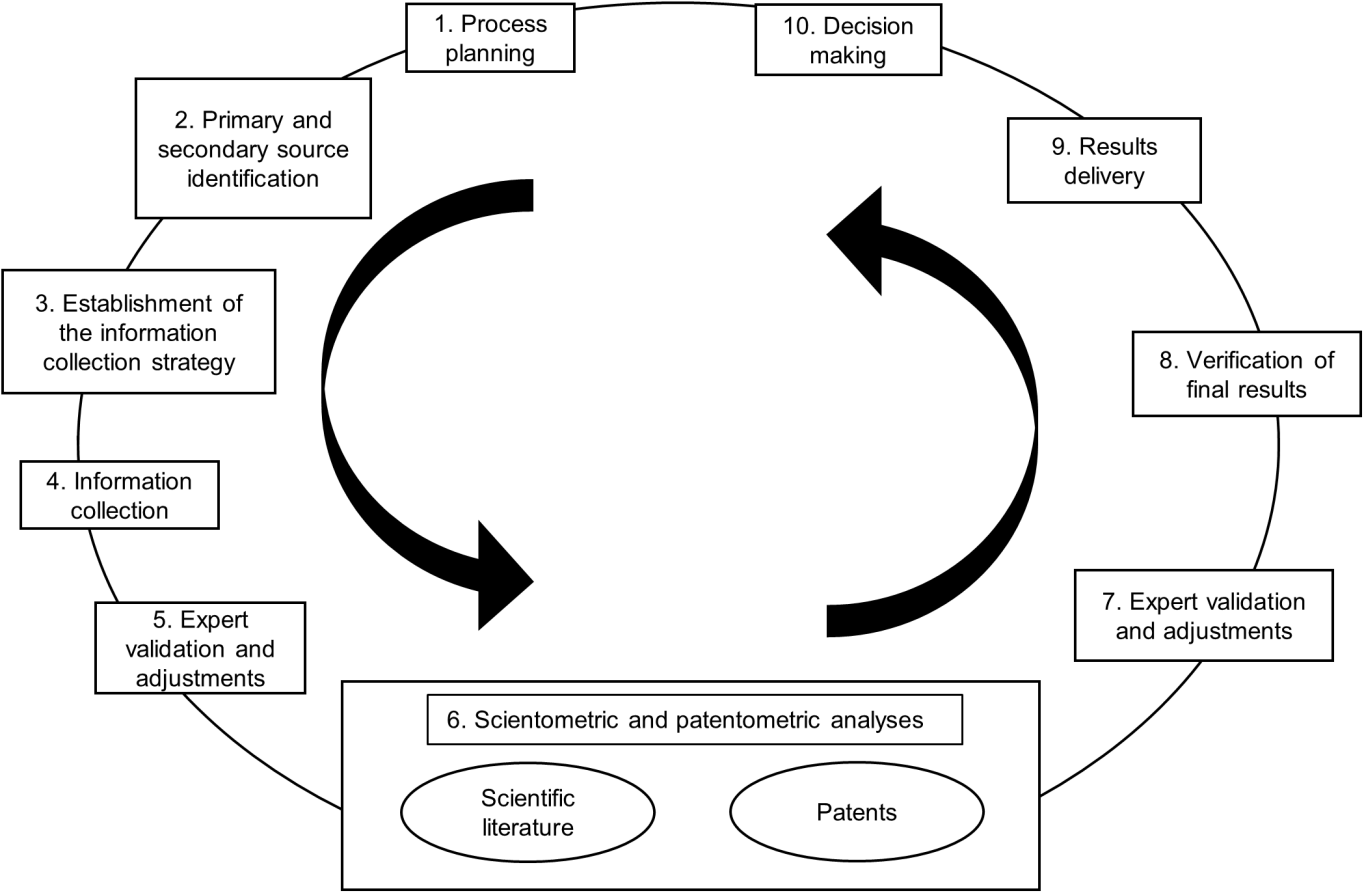
Competitive technology intelligence (CTI) and expert perspective hybrid data model. This flow chart outlines the 10 main steps of the methodology implemented in the present study. The steps are indicated in boxes and sub-steps are indicated in ovals. The methodology begins with step one (the planning process) and continues through step 10 (decision making). The steps are repeated iteratively until the desired result has been obtained.

The approach used in this study was developed as a cyclical process, which is rooted in the established CTI methodology and comprises 10 main steps that are shown in Fig 1. The methodology starts with a planning process where the main goals, activities, participants and the allocation of resources are stated. Normally, the party responsible for R&D is also the end user of the CTI project, thus the goals must align in content and style with the organization’s needs. The process of development of a CTI activity requires a full understanding of the intelligence required by the final user and how this intelligence should be delivered [29]. The second stage consists of the identification of primary and secondary information sources and further source selection. Primary information comes from experts who are selected by criteria such as their professional position, number of academic citations, number of highly ranked publications or general presence in the field. Meanwhile, secondary sources, such as databases and reports, are also analyzed with regard to their quality and prestige, coverage and completeness of information. The next two steps are the definition of the information collection strategy and the gathering process. These include the determination of the most suitable queries to be used in retrieving information from databases and their subsequent implementation. This is a fundamental step and involves expert consultation from the conception through the validation of results. The analysis stage follows. This should implement advanced text mining software that has access to major scientific and patent databases, as well as advanced analytical capabilities to process thousands of documents to facilitate the determination of the evolution of the field and its dynamics. Co-occurrence and keywords clusterization techniques are a key part of this task, and has been used [30] to distinguish the main research areas of a given field.

Next, expert assessment is used to evaluate the search strategy definition, validate the information collected and review the analysis stage, which commonly require ratification and adjustments before arriving at the final results. Insights obtained should be relevant enough to shed light upon competitive opportunities and threats [5]. Moreover, results should be delivered according to specific—and previously defined—goals and requirements (for example, time, format and technical language) demanded by the final customers, in such a way that they can complete the CTI cycle by making valuable decisions based on the resulting data.

### Application of the proposed model to 3D bioprinting

The process described in Fig 1 (see corresponding numbers in parentheses) was implemented as is explained in the following sections. It began with the process planning stage (1), which established the activities of the study required to accomplish the main objective, based on this an identification of information sources (2) was made covering primary (expert) and secondary (scientific literature and patents databases) sources. After that, the information collection strategy (3) including specific queries for databases were stated. Information collection (4) involved an extensive literature review on 3D printing, 3D bioprinting and tissue engineering. Additionally, consultations with experts (5) in both academia and industry were performed to validate results. The scientific literature and patents found were then subjected to a scientometric and patentometric analysis (6), which was again validated by expert feedback (7). The final results were then verified (8) and adjusted for delivery (9) aiming to contribute the decision making process (10).

#### Expert collaboration

Experts in the field of the technology being studied should be involved in the analysis. For this case, top researchers in 3D bioprinting were identified in the UK. Locally-based experts were selected primarily from the top 3D bioprinting research groups at the University of Manchester and the University of Nottingham, based on their international presence in the field. Qualitative interviews were conducted in person. The University of Manchester has a long and distinguished history in the development of 3D bioprinting. An early use of the term “bioprinting” is found in the title of the ‘Workshop on Bioprinting, Biopatterning and Bioassembly’ held at this university in 2004 [31]. Over the last several decades, researchers from the University of Manchester have devoted significant efforts to the advancement of the field, including new bioink technologies and bionano research. Experts were selected from both the School of Materials, and the School of Mechanical, Aerospace, and Civil Engineering at the University of Manchester. These experts—who develop research on 3D bioprinting—were interviewed multiple times from the early stages of the research to the final results validation. In particular, two researchers were consulted, as they have more than 6600 and 1500 Scopus citations, respectively, on subjects such as biomaterials, cell and tissue engineering, cell adhesion, manufacturing of functional components, organ printing, and bone and cartilage regeneration.

In addition, an important center that aims to influence the worldwide 3D printing research agenda was identified during the early stages of this research. This is the Engineering and Physical Sciences Research Council Centre for Innovative Manufacturing in Additive Manufacturing, which has been hosted by the University of Nottingham in partnership with Loughborough University since 2012 [32]. This center is developing research that is moving toward a new generation of additive manufacturing: multifunctional manufacturing, where multiple dissimilar materials can be printed within a single process. Along this theme, 3D bioprinting can go beyond the deposition of individual passive components to print entire integrative working systems [33]. Here, many different projects relating to 3D bioprinting are taking place simultaneously.

Four researchers from the 3D bioprinting research groups at the University of Nottingham were interviewed. They belong to the Faculty of Engineering and the School of Pharmacy (Center for Biomolecular Sciences), two of them have more than 760 and 280 Scopus citations, respectively, on topics related to the generation of micro-structures for tissue engineering, regenerative medicine, coatings for implants, 3D printing of human tissue, drug delivery system, and material-cell interaction, among others.

During all of the steps outlined in Fig 1 experts from both the University of Manchester and the University of Nottingham provided feedback, predominantly on the analysis and interpretation of scientific documents and patents, including the final validation step.

#### Search query definition

The definition of a proper search query is crucial especially due to the fact that emerging technologies encompass different approaches. This task was carried out through an iterative process whereby different terms and queries were tested by collecting information from databases and validating the results with experts, as described above. It is also important to select databases that broadly cover the emerging technology. This study used the most comprehensive databases available, as is shown in the next section,.

**Fig 2:**
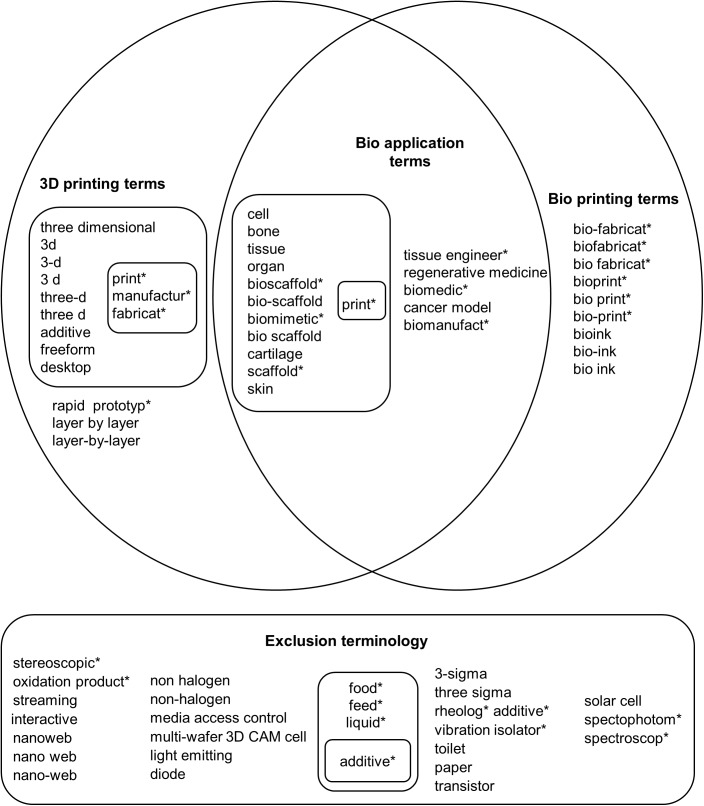
Main query terminology for the database searches. Words marked with an asterisk (*) are root words, indicating that all possible suffixes are covered under the query.

In order to build a search query for 3D bioprinting, we determined specific terms that occur within the field according to three main groups, as is shown in Fig 2. They are 1) the 3D printing process itself (3D printing terms), 2) the bioprinting process (bioprinting terms) and 3) the biological words that represent fundamental applications of 3D bioprinting (bioapplication terms). Previous work by Groll and colleagues [33] on the definitions used in the biofabrication field across scientific disciplines, such as 3D printing, was incorporated. Exclusion terms were defined based on reports in 3D bioprinting, expert consultations and manual inspections during query trials.

Each term was tested in different query structures in both the scientific and patent databases. The purpose of this was to identify variations and the pertinence of the information retrieved. Expert participation was integrated continuously during this activity. Finally, a global query was determined and is listed in S1 Appendix (‘Global Query’).

Variations on the query are often necessary due to syntax specifications of the different databases. To delimit the starting year of the information collected from the databases, a deep literature revision as well as expert consultations should be carried out. In this research we identified that the first reports of 3D bioprinting were made in early 2000 [34], while “organ printing” terms referring to the use of 3D printing first appeared in 2003, and the first use of the term bioprinting was in 2004 [33]. Based on this, 2000 was defined as the initial year for the gathering stage and 2016 (until July 1^st^ when the information-gathering component of this research concluded) as the final year.

#### Scientific literature data collection

The Scopus and Web of Science (WoS) databases were selected to be used as the primary search engines in this study, as they have the most extensive coverage of published scientific papers. Scopus is one of the largest abstract and citation databases of peer-reviewed science and technology literature. It provides access to more than 5000 publishers worldwide [35]. Likewise, WoS is a database that groups publications from over 7000 academic and research institutions, including governments and organization in over 100 countries [36]. As Burnham [37] establishes, both databases are powerful tools and are complementary as “neither resource is all inclusive”. We recommend these databases for further CTI studies on emerging technologies.

The main query, defined in the previous section (‘Global Query’ in S1 Appendix), was adjusted to be used in each of these databases. For this task, a process that included adaptation to the database-specific syntax and manual validation of the results, as well as expert consultation, was carried out to determine accuracy. The resulting queries are listed for the respective databases in S1 Appendix (‘Scopus Query’ and ‘WoS Query’). The Scopus query recovered a total of 2634 articles and conference proceedings between January 1st, 2000 and July 1st, 2016. A total of 2148 journal articles and conference proceedings were retrieved for the same period from the WoS database. The Scopus and WoS exported data can be found in S1 File and S2 File, correspondingly.

Scientific literature data (journal articles and conference proceedings) obtained were mined with Patent iNSIGHT Pro, a software platform that incorporates advanced text mining algorithms [38]. A total of 4782 documents (Scopus and WoS) published between January 1st, 2000 and July 1st, 2016 were collected, and then followed an extensive cleaning and deduplication process that retained 4723 unique documents. These data underwent a normalization process which consisted of treating synonyms and acronyms as the same word. A “fuzzy match” algorithm present in the software enabled the identification of similar keywords and assignee names with 85% coincidence. Specific groups were automatically built based on this criterion. A further validation of the suitability of each grouping was made through manual inspection and incorporating experts’ feedback.

#### Patent collection

This activity was carried out using PatSeer [38], a software program designed for research, analysis and project management that permits access to more than 92 million records from the main patent offices worldwide. It covers bibliographic data from 140 countries and the full text of 27 authorities including Espacenet (EP), the World Intellectual Property Organization (WO), the United States Patent and Trademark Office (US) as well as the patent bodies of Japan (JP), China (CN), Korea (KR), Canada (CA), Germany (DE), France (FR), Great Britain (GB), Spain (ES), Australia (AU), India (IN), Switzerland (CH), Austria (AT), Brazil (BR), Thailand (TH), Russia (RU), Philippines (PH), Sweden (SE), Norway (NO), Denmark (DK), Finland (FI), Belgium (BE), Netherlands (NL), Luxembourg (LU) and Mexico (MX).

As with the scientific literature data search described above, an adaptation of the Global Query (S1 Appendix) was made to search this patent database and a validation of the results was conducted manually. Patents were searched according to title-abstract-claims (TAC) where the title and abstract describe the main characteristics of the patent, while the claims section focuses on how the invention was developed. The final query is listed in S1 Appendix (‘TAC Query’).

A total of 706 documents were found that cover patent applications and grants in 3D bioprinting filed between January 1st, 2000 and July 1st, 2016. These patents are contained in S3 File. A deduplication process was carried out to eliminate repeated documents. This reduced the total number of documents to 601. In order to improve the research accuracy, patents were grouped by families, resulting in 345 patent families (PFs). The European Patent Office [39] defines PF as “a group of either patent applications or publications made in multiple countries to protect a single invention by a common inventor(s)”. The first application is made in one country—the “priority country”—and is then extended to other offices, thus creating a PF.

## Results and discussion

In this section, the results obtained through the applied CTI data model are presented.

### Scientific publication trends in 3D bioprinting

**Fig 3:**
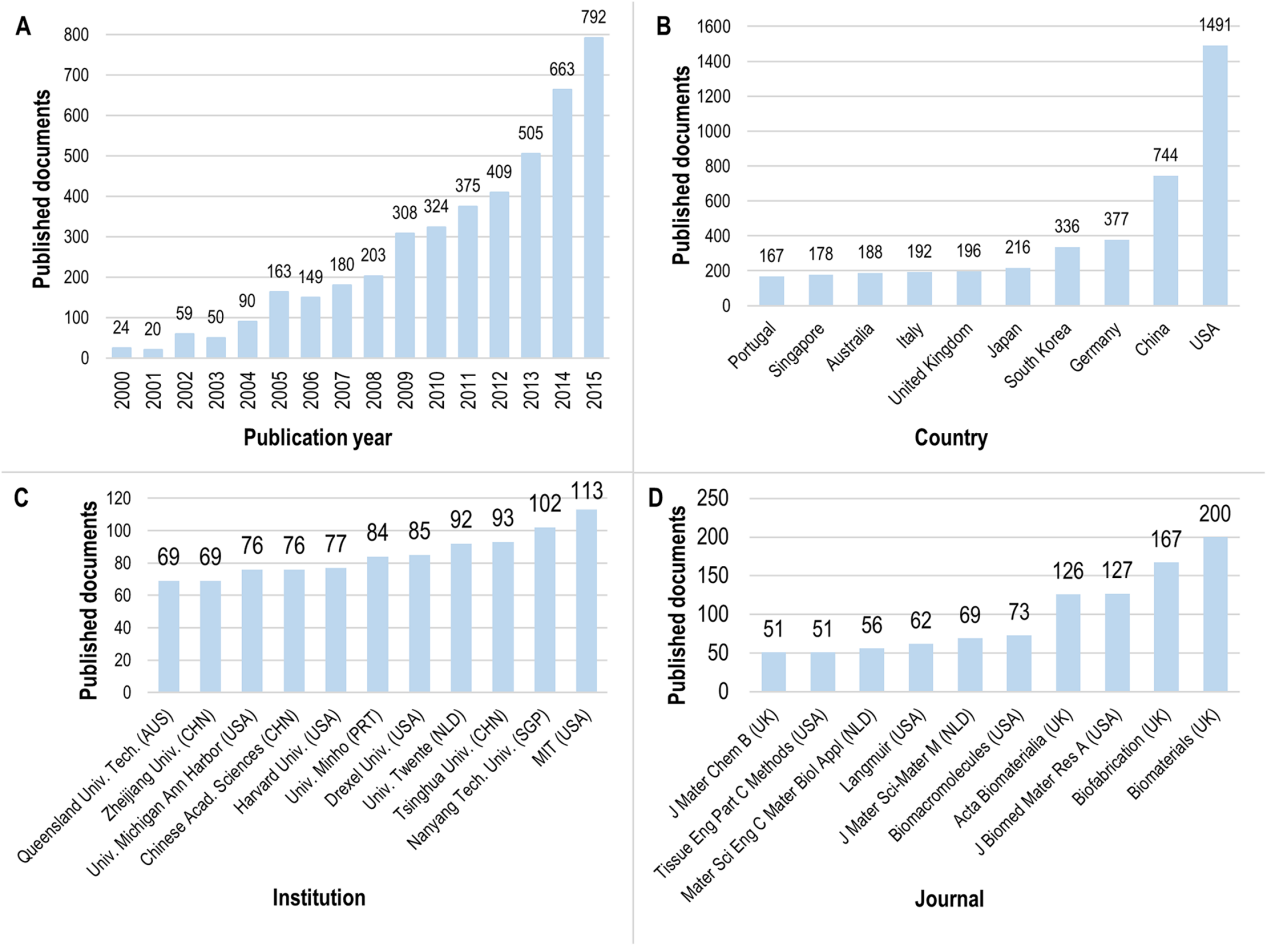
Global scientific trends in 3D bioprinting. A summary of the publications on 3D bioprinting that are indexed in Scopus and the Web of Science according to (A) publication output by year, from 2000 to 2015; (B) the 10 most frequent affiliation countries of the authors; (C) the 10 most frequent organizational affiliations of the authors (11 institutions are reported due to a tie for tenth place); and (D) the 10 journals with the most occurrences of the search terms.

Scientific knowledge production in 3D bioprinting since the beginning of 2000 was determined from the results of the corresponding Scopus and WoS analyses: the results are shown in Fig 3A–3D. The year 2016 is not included in Fig 3A as the results (409 documents) only represent a partial year (January 1 through July 1). Global results show that 3D bioprinting is rapidly gaining attention in R&D, which in turn impacts scientific production: particularly in the most recent years. While in 2000 there were only 24 scientific publications relating to 3D bioprinting, this number rose to 792 in 2015, an increase of 3300%. Of the 4314 scientific documents on 3D bioprinting published from 2000 to 2015, 71% were published in the last five years (2010–2015), showing the novelty and increasing interest in 3D bioprinting. Within this same period, there was an annual average growth rate in publications of 20%. In the most recent years, there was a publication increase of 31% from 2013 to 2014 and an increase of 19% from 2014 to 2015. An exponential regression was done based on the data from 2006 (the year in which the publication trend began to increase continuously) through 2015. The equation describing the data was y = 127.13e^0.1801x^ with a coefficient of determination R^2^ = 0.9823. If the publication increase continues at the same rate, the equation forecasts that 921 documents will be published in 2016 and 1894 in 2020.

The affiliations of authors of scientific papers indexed in high-impact scientific databases (such as Scopus or WoS) are an indicator of which countries and organizations have specific patterns of research concentration and excellence [40]. Here, predominant countries and organizations in the field of 3D bioprinting are presented. The 10 most prolific nations in terms of publishing in 3D bioprinting (shown in Fig 3B) are not geographically concentrated. More than 37% of all publications (1491 documents) come from the USA, and it published over twice as many documents as the following country, China (744). Likewise, China has close to double the number of publications as the next most prolific nation, Germany (377). The remaining countries in the top 10 each published between 167 and 336 documents and are all located in either western Europe or eastern Asia—with the exception of Australia, which holds the eighth position.

The top publishing institutions (shown in Fig 3C) are highly correlated with the top 10 countries, as most of these institutions are American (4) and Chinese (3). However, the rankings in this category are much closer in their total output, which indicates that competition is more active. The top three institutions are the Massachusetts Institute of Technology (USA, 113 documents), Nanyang Technological University (Singapore, 102) and Tsinghua University (China, 93). The institutes with the next highest levels of output follow closely; top organization rankings could vary greatly in the coming years.

Scientific development in 3D bioprinting has led to the creation of new journals focused on the topic, and consequently the number of scientific publications has increased. An analysis was made to identify the most prolific 3D bioprinting journals in terms of the volume of documents published in the field. The top 10 most prolific journals are shown in Fig 3D. Journals with over 100 published articles or conference proceedings relating to 3D bioprinting are summarized in Table 1. All four of these journals are indexed in the Thomson Reuters Journal Citation Report [41] under the “engineering, biomedical” and “material science, biomaterials” categories, with upper quartile rankings.

**Table 1:** Journals with more than 100 articles or conference proceedings on 3D bioprinting.

Position	Journal	Year of creation	3D bioprinting articles	JCR 2015 impact factor	JCR categories ranking			
					Engineering, Biomedical	Quartile	Material Science, Biomaterials	Quartile
1	Biomaterials	1980	200	8.387	2/76	1	1/33	1
2	Biofabrication	2009	167	4.702	6/76	1	5/33	1
3	J. Biomedical Materials Research Part A	1967	127	3.263	13/76	1	14/33	2
4	Acta Biomaterialia	2005	126	6.008	3/76	1	2/33	1

Position is according number of articles published on 3D bioprinting. The Journal Citation Report (JCR) impact factor is the two-year index according to the 2016 JCR citation report.

### Patent trends in 3D bioprinting

**Fig 4:**
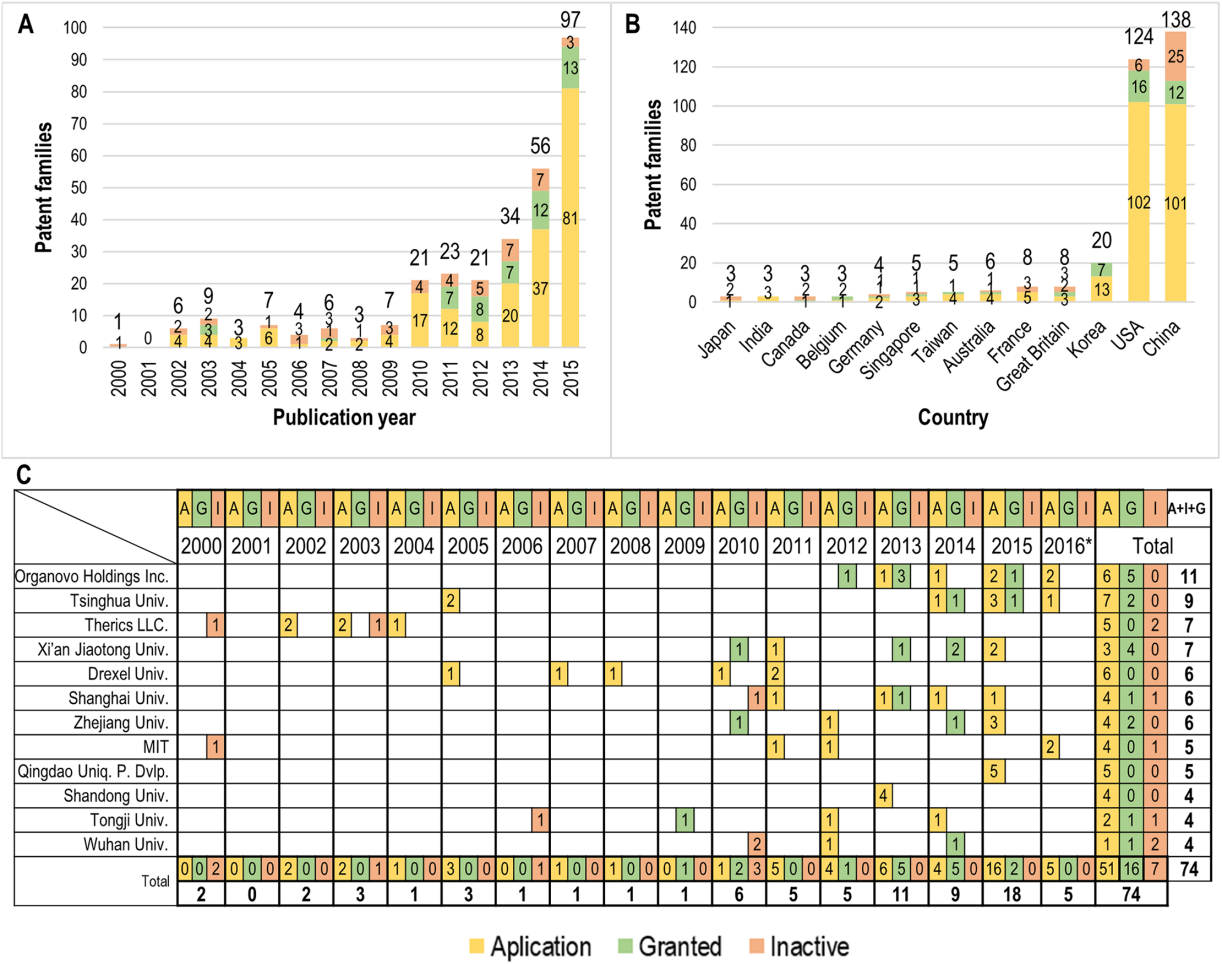
Publication rates and geographical distribution of 3D bioprinting patents. (A) The number of 3D bioprinting patent families (PFs) by year from 2000 to 2015. (B) The number of 3D bioprinting patents applied for in each of the top 10 most prolific priority countries (i.e. countries in which the first patent of a PF was applied for). Four countries (Belgium, Canada, India and Japan) were tied for the tenth position. (C) The number of PFs ranked according to assignee institutions by year, from January 1, 2000 until July 1, 2016. Three institutions (Shandong University, Tongji University and Wuhan University) were tied for the tenth position. In all three graphs, yellow indicates grants that were applied for, green indicates PFs that were granted, and red indicates inactive PFs.

The patent trends, the patent priority countries and a timeline of patents of the top 10 patent assignees from January 1st, 2000 to July 1st, 2016, along with the patent’s current legal status (assigned, granted or inactive), are presented in Fig 4A–4D. The patents obtained were grouped into PFs, according to the protocol described in the methods section. Text mining software was used to collect information and followed a similar deduplication, cleaning, normalization and validation process as was implemented for the scientific literature. In addition, every PF was manually examined and individually categorized as a patent application, granted patent or inactive patent. In this period, a total of 345 PFs were detected, with an overall application-granted-inactive patent proportion of 70%-17%-13%. Due to the novelty of this technology, most of the patent applications have not been granted yet.

For the period from 2000 to 2015, 298 PFs in 3D bioprinting were detected and 47 PFs (41 applications, 6 granted, 0 inactive) were found for the first half of 2016. The results in Fig 4A show rapid growth in the number of patents in this technology. While in 2000 there was only one PF and none in 2001, the number rose to 97 in 2015, corresponding to an increase of 9700%. An annual average growth rate of 40% was detected from 2010 to 2015. Moreover, this period contains more than 84% of all PFs. Results obtained show a consistent increase over the last several years: 65% from 2013 (34 PFs) to 2014 (56 PFs) and 73% from 2014 (56 PFs) to 2015 (97 PFs). As with the 3D bioprinting publication rate, an exponential regression was created to describe the rate of patent growth from 2006 to 2015. The resulting equation was y = 2.1429e^0.3628x^ with a coefficient of determination R^2^ = 0.9023. If this trend continues, significant growth is forecasted for 2016 and 2020, with the number of annual PFs reaching 115 and 494, respectively.

In order to determine the nations of origin of 3D bioprinting PFs, the main countries where applications were first filed before being (possibly) extended to other countries were identified. China dominates with 138 PFs during this period (2000-July 1st 2016), as shown in Fig 4B. It holds an application-granted-inactive ratio of 73%-9%-18%, making it also the country with the most inactive PFs. The second most PFs come from the USA, with a total of 124 and an application-granted-inactive ratio of 82%-13%-5%. Thus, if the inactive PFs were discarded, the USA would outpace China with 118 vs. 113 PFs, which resembles the results shown in Fig 3B where the USA ranks highest in terms of total scientific publications on 3D bioprinting. South Korea has the third most PFs, with 20, and an application-granted-inactive ratio of 65%-35%-0%. All other countries have produced less than 10 PFs. The overall application-granted-inactive ratio for the top 10 priority countries is 73.3%-13.3%-13.3%. The most significant outliers for granted and inactive PFs are the USA, which holds 36% of all granted PFs, and China, with 57% of all inactive PFs.

The top patent generating organizations and their patent activity through time are presented in Fig 4C. Organovo Holdings, Inc. (USA) has the most PFs (11 PFs, of which 6 are applications, 5 granted and 0 inactive). This is consistent with results from the literature and expert consultations, where Organovo was identified as the leading company in 3D bioprinting. Founded in 2007, this company designs and creates functional human tissues. It was founded with the support of patented technologies from the University of Missouri (USA) [42]: Organovo’s patenting activity in 3D bioprinting was detected in this research from 2012.

Tsinghua University (China) follows with 9 PFs (7 application, 2 granted, 0 inactive), with its first patent activity during the period assessed found in 2005 and, after years of inactivity, it recommences patent applications in 2014. Founded in 1911, Tsinghua University has become one of the biggest and most outstanding Chinese universities. In December 2015, it had 20 schools and 54 departments “with faculties in science, engineering, humanities, law, medicine, history, philosophy, economics, management, education and art” [43–44] This university has recently risen to a top position in R&D in 3D bioprinting, as the patent and scientific publication analyses indicate (Figs 2C and 3C).

In the third position both Therics, LLC. (USA) and Xi’an Jiaotong University (China) have 7 PFs. Therics produced bone substitutes and was founded in 1996 as a subsidiary of Tredegar Corporation [45]. Therics began its patenting activity early in the period analyzed: between 2000 and 2004, 7 PFs are found, of which 5 are applications and 2 are inactive. After that, patenting efforts ceased and in fact the applications made were never granted. The main reason for this could be that in 2008 the company was acquired by Integra Life Sciences, a company that develops solutions for orthopedic extremity surgery, neurosurgery, and reconstructive and general surgery [46], but does not work with 3D bioprinting. Xi’an Jiaotong University also has produced 7PFs, although its rate is different with 3 applications and 4 granted patents. Founded in 1896, this university is an institution focused on the advancement of science and engineering in a number of fields including new ones such as 3D bioprinting [47]. With patenting activity first identified in 2010, Xi’an Jiaotong University is the assignee with the second most granted patents (4, just below Organovo that has 5), and it currently has no inactive patents.

### Global technology trends

Global trends for the countries reveal a prominence of the USA and China in scientific literature and patent production in 3D bioprinting. The USA has a long tradition of strong investment in R&D. In the last 20 years, it has increased its R&D expenditure from a 2.4% to 2.7% of its gross domestic product (GDP). Globally, it is the current leader in net investments in R&D, with 28% of the global expenditure, reaching a total of $457 billion US dollars [48]. This clearly has produced a significant effect on the development of breakthrough technologies, such as 3D bioprinting. However, according to [49], China is rapidly approaching the USA, and is forecasted to outpace the country in total expenditures in R&D by 2019. Currently, China ranks second with 20% of the global expenditure ($369 billion US) on R&D [48].

China’s leading position in science and technology can be explained as a result of specific government actions implemented over the last decade. In 2006, the Chinese government established a 15-year medium-to long-term plan for the development of science and technology with the goal of rising to the position of an “innovation oriented society” by 2020 and to become a world leader in science and technology by 2050. According to this plan, China will invest 2.5% of its increasing GDP in R&D by 2020, up from 1.34% in 2005. Additionally, it aims to become a top-five country in terms of patenting and most cited papers [50]. As a result of this government action, China doubled the number of patents it produced in the period from 2007 to 2012. They also increased their R&D general budget, enforced tax breaks, promoted investments in academic institutions and enhanced monetary incentives [51].

The results presented here have shed light on how 3D bioprinting is attracting the attention of industry and academia. Scientific and technological research is growing exponentially thanks to the efforts of large nations such as the USA and China, among others.

**Fig 5:**
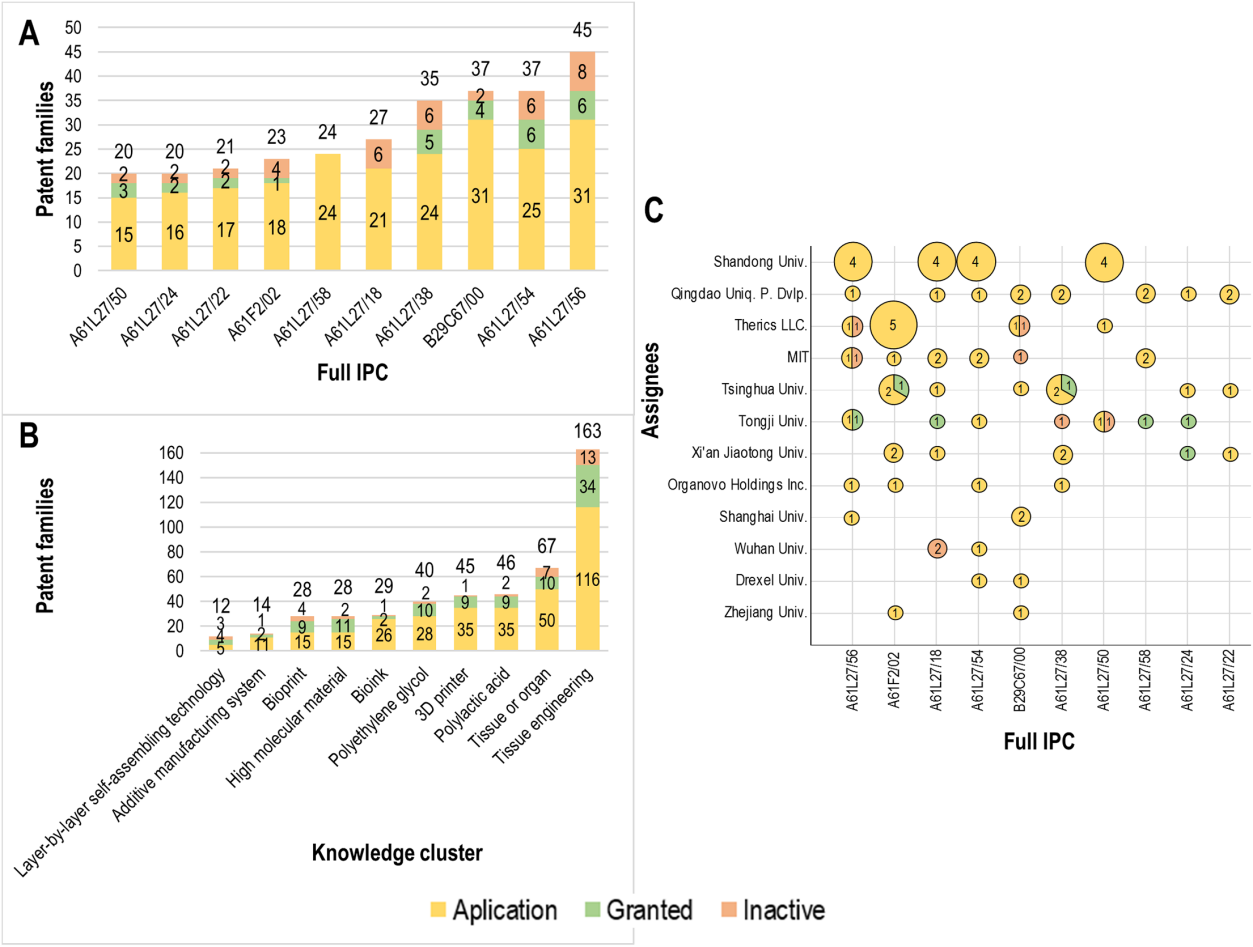
Global technology trends in 3D bioprinting. (A) The top 10 International Patent Classification (IPC) classes in which 3D bioprinting patent families (PFs) identified in this study are found. (B) The top 10 “knowledge clusters” for IPC 3D bioprinting patents since 2000. The knowledge clusters were developed using a clustering algorithm based on unique terms occurring in the title, abstract and independent claims of patents. (C) The IPC classes of patents by each of the top assignees identified in Fig 4. Yellow indicates PFs that have been applied for, green indicates granted PFs and red indicates inactive PFs. It is important to note that each patent may be listed under several IPC classes, which is why the total number of PFs in (A) and (B) are greater than the number of PFs presented in (C).

An analysis to determine where R&D efforts in 3D bioprinting are focused was developed in light of patent areas concentration. For this task, the top International Patent Classification (IPC) patent codes, knowledge areas of research (keyword clusters), and the orientation of technology efforts of organizations (assignee-IPC patent code co-occurrence) were determined, as is shown in Fig 5A–5C.

The IPC is an international classification system that provides standard information to categorize inventions and to evaluate their technological uniqueness [52]. IPC classes enabled the identification of the main foci of 3D bioprinting inventions. Results show that the most common IPC section for 3D bioprinting patents is materials for (grafts or) prostheses or for coating (grafts or) prostheses (A61L27). Within the top 10 classes shown in Fig 5A, 45 PFs relate to porous or cellular materials (A61L27/56), 37 PFs deal with biologically active materials, e.g. therapeutic substances (A61L27/54) and shaping techniques not covered by other groups (B29C67/00), 35 PFs concern animal cells (A61L27/38), 27 PFs refer to macromolecular materials obtained by means other than by reactions only involving carbon-to-carbon unsaturated bonds (A61L27/18), 24 PFs deal with materials that are at least partially resorbable by the body (A61L27/58) and 23 PFs relate to prostheses implantable into the body (A61F2/02). The application-granted-inactive ratio is similar to that seen in Fig 4 with 77%-10%-13%, respectively.

In order to complement the summary shown in Fig 5A, a clusterization of keywords was carried out to identify areas where global research in 3D bioprinting is the most active (Fig 5B). The clusters were produced by extracting raw keywords from the title, abstract and independent claims of patents and then merging synonyms, eliminating noisy terms, and categorizing the resulting terms using an algorithm that scans and clusters single concepts in a multi-level hierarchy [53]. Each cluster represents a unique concept related to 3D bioprinting, according to the occurrence of keywords. Therefore, similar terms will not necessarily be placed in the same cluster. Grouping will only occur when the terms share a high co-occurrence.

Fig 5B shows that the terms within the major knowledge clusters are: tissue engineering (163 PFs), tissue or organ (67), polylactic acid (46), 3D printer (45), polyethylene glycol (40), bioink (29), high molecular material (28), bioprint (28), additive manufacturing system (14) and layer-by-layer self-assembling technology (12). Furthermore, there are clusters that we consider relevant but that did not reach the top 10. These included alloy powder (10), which encompasses the terms “titanium alloy” and “porous structure”; double molecule layer (8), which comprises chemical compounds such as “ethylenediamine” and “chlorosulfonic acid”; microcapsule (7), which involves chemical terms such as “layers of polypeptides”, “sodium alginate”, “acid copolymer” and “CaCl_2_”; and imaging systems (5), which contains terms such as “laser beam”, “magnetic resonance” and “imaging systems”.

The clusters with the highest granted PFs ratio are high molecular material (39%), layer-by-layer self-assembling technology (33%) and bioprint (32%). This is notable as the average number of granted PFs in the top 10 clusters in Fig 5B is only 21%. These outliers are likely to indicate that these aspects of 3D bioprinting technology have flourished. Table 2 shows the terms included within each of the 10 knowledge clusters presented in Fig 5B.

**Table 2:** Knowledge cluster breakdown.

*Knowledge cluster title*											
		Tissue engineering	Tissue or organ	Polylactic acid	3D printer	Polyethylene glycol	Bioink	High molecular material	Bioprint	Additive manufacturing system	Layer-by-layer self-assembling technology
*Constituent terms*											
	• 3D printing • Acid anhydride • Alkyl and R • Antimicrobial agent • Bio-ink at a ratio • Biological reactor • Blood vessel • Bone tissue engineering • Branched alkyl • Chemical or biological agent • Coatings for biomedical devices • Composite device • Crystalline polymer • Degree of polymerization • Growth factor • Mesenchymal stem cells • NH 4 • Polystyrene • Plurality of cell aggregates • Poly glutamic acid • Polyelectrolyte multilayers • Porous metal bracket • Porous polymer membrane • Portion of the wall • Self-assembly technique • Sodium hydroxide solution • Solid matrices • Supernatant fluid • Tissue engineered cartilage • Tissue engineering nerve • Tissue engineering scaffold • Tissue engineering supporting • Tubular epithelial cells • Unsubstituted aryl		• 3D printing • Bionic structure • C. 5 • Cell aggregates • Cell array • Electromagnetic force • Electromagnetic radiation • Glycolic acid copolymer • Holding vessel • Hydroxyl acetic acid • Inkjet printing • Lactic acid • M sodium chloride • Muscle cells • Nerve blood vessel • Plurality of pores • Polycaprolactone film • Porous ceramic • Progenitor cells • Stem cells • Thin film • Three-dimensional mesh	• 3D object for printing • Artificial vascular stent • Blood vessel bracket • Bone morphogenetic protein • Cell toxicity • Core layer • Culture medium • Hole clearance ratio • LbL film • Light source • Lower part • Material ink • Natural biological material • Nerve nutrition gene • Polyester polyurethane • Polylactic acid film • Polymer component • Rapid forming technology • Repair scaffold • Shape memory • 3D inkjet printing • Tissue regeneration • Wound dressing	• 3D biological printing • 3D printing machine • Additional layer • Average diameter • Biological supporting bracket • Bone growth factor • Consisting of a mixture • Ethyl alcohol • Means of 3D printer • Output device • Polymers and copolymers • Polyvinyl alcohol • Porosity of 90% • Regenerative medicine • Skin graft product • Support structure • 3D print technology • Tissue 3D printing • Three-dimensional printer • Tissue engineering blood vessel • Tissue engineering skin	• Buffer solution • Composite scaffold • Cross linking • Dimensional array • Fiber layer • Flow passage structure • Functional groups • Hydrogel material • Implantable device • Manufacturing a ceramic • Measuring tissue • Natural macromolecule solution • Print head • Production process • Soft tissue • Step of bioprinting • Tissue repair • Vascular endothelial cell • Vascular graft • Water gel solution	• Acetic acid • Adhesive layer • Biodegradable material • Cells per ml • Connective tissue • Cross-linking reaction • Endothelial cells • Ethylene glycol • Layer thickness • Polyethylene glycol • Printing machine • Support material • Tissues and organs • Tumor model • Tumor tissue • UV light	• 3D printing porous metal • Artificial blood vessels • Base material • Biological printing • Bone defect part • Cell growth factor • Composite porous metal • Dimensional slightly bracket • Fuse extrusion system • Scaffold material	• Amino acid • Biocompatible polymer • Biologic printing system • Desired shape • Electronic device • Follicle stem cells • Good biocompatibility • Image data • Ink jet • Living cells • Multicellular body • Nozzle assembly • Organic solvent • Particle feeding mechanism • Single shaft step motor • Printing object • Three-dimensional model	• Ceramic material • Computer-aided design • Template method • Data file • Device and control • Dimensional printing method • Print foundation • Drive mechanism • Printing three-dimensional parts • Printing axis • Three-dimensional structure • Tissue scaffold	• Active component • Blood cell • Multiple layer film • Polylactic acid • Polyurethane material • Multilayer film • Polyurethane film • Solution to dip • Surface modification method

Terminology included within each of the 10 knowledge clusters presented in Fig 5B. Each knowledge cluster was developed by extracting keywords from the title, abstract and independent claims of patents, cleaning the data and then clustering using an algorithm.

In order to fulfill the final steps of the hybrid data model, validation of the results and further adjustments were made with the support of the previously described experts from the University of Manchester and the University of Nottingham. Insights revealed that research on 3D bioprinting is mainly driven by eight key elements. These are depicted in Table 3, where a direct linkage with the previously obtained knowledge clusters is established.

**Table 3:** Linkage between 3D bioprinting drivers identified by experts and knowledge clusters generated through text mining software.

Drivers identified by experts	Description	Knowledge Cluster	
		Position according to their number of patent families	Cluster
Tissue engineering	Tissue and organ repair, maintenance or replacement through printing techniques	1	Tissue engineering
		2	Tissue or organ
3D bioprinting systems	Devices to print molecules, cells, tissues and biodegradable materials	4	3D printer
		9	Additive manufacturing system
Bioinks	Development of new biological, biocompatible and bioabsorbable materials (polylactic acid, polyethylene glycol and others) that can be printed while maintaining integrity and structure, and keeping cells alive	6	Bioink
		3	Polylactic acid
		5	Polyethylene glycol
		10	Layer-by-layer self-assembling technology
Fibers and scaffolds	Development of fundamental structures through 3D bioprinting	7	High molecular material
		5	Polyethylene glycol
Human body models	Development of models for a better understanding of human body behavior, including mechanisms that elicit diseases, as well as those that are part of their prevention and treatment	6	Bioink
		8	Bioprint
Regenerative medicine	Advancements in regenerative medicine, especially the study of cancer	4	3D printer
Pharmaceutical research	Pharmaceutical research regarding drug dosage modes, delivery and discovery	17	Anatomical body part
Vascularization (development of blood vessels)	Development of new ways to repair and maintain vascularization of tissues or organs	1	Tissue engineering
		2	Tissue or organ
		3	Polylactic acid
		4	3D printer
		7	High molecular material

It is important to point out that clusters result in more focused topics compared to the drivers identified by experts. For example, bioink technology is grouped as a single driver by the experts, while its components are scattered in different knowledge clusters including bioink, polylactic acid and polylethylene glycol. This is likely to indicate that an original technology (bioink) comprises other advancements (polylactic acid and polylethylene glycol). In addition, clustering differences between experts and text mining approaches reveal how emerging technologies might be categorized in different ways.

This hybrid approach results in an effective method to validate results. Every top 10 knowledge cluster generated by data mining was contained within the identified drivers by experts, except for “pharmaceutical research”, a component found in the knowledge cluster ranked 17th as an “anatomical body part”. This knowledge cluster contains the following elements:

- Radiological data
- Active pharmaceutical ingredient
- Magnetic resonance imaging
- Rapidly customizing design
- Successive multi-dimensional digital models

The research organizations’ foci were determined by assessing the top 10 assignees’ co-occurrence with the top 10 IPC classes (Fig 5C). This shows that the most frequently co-occurring top IPC classes are porous or cellular materials (IPC code A61L27/56), prostheses implantable into the body (A61F2/02) and macromolecular materials obtained by means other than by reactions only involving carbon-to-carbon unsaturated bonds (A61L27/18). Of the top 10 assignees, the ones that have the most PFs within the top 10 IPC classes are: Shandong University (China, 16), Quingdao Unique Product Development (China, 12), Therics, LLC. (USA, 10), Massachusetts Institute of Technology (USA, 10) and Tsinghua University (China, 10). This could indicate that these assignees publish the most focused patents within the top technological IPC classes. However, the applications-granted-inactive ratio is 83%-8%-9%, which reveals that the vast majority of the PFs found in the top assignee/IPC class co-occurrence analysis are recent (in the initial application state).

The data show that 3D bioprinting is still in an early stage and that interest from academia and industry is evolving and increasingly growing. Given the fact that the patenting process takes years, only a small number of applications have been granted thus far. In light of these insights, the results delivery phase can be pursued (Fig 1). It is important to put forward the results of the goals defined in the initial stage. This includes presenting the content according to the style defined by the end user: normally the person involved in R&D decision-making. Conciseness, opportunity and relevance are key aspects of this stage. An executive report can be produced that condenses the main insights obtained.

## Conclusions

This research presents a novel approach to define the knowledge landscape for emerging technologies. A hybrid data model that combines scientific and technological output analysis and expert feedback was built. We encourage CTI researchers and professionals to apply this model for the study of innovative technologies with disruptive potential.

This model was implemented on the ongoing scientific and technology research in 3D bioprinting. Several reports [12, 14, 54] indicate that this is a promising technology that will revolutionize the health industry as it enables the repair, replacement or control of functions within or on the human body. Moreover, it is expected that by the late 2020s [54] this technology will be used to print complex organs, resulting in a sustainable alternative to organ donations.

Blending scientometrics and patentometrics with experts’ insights brings numerous advantages as a CTI approach. It is a symbiotic process and when properly executed, takes the best of both methods. A more robust process could be developed along all the stages of the model, including validation from the early stages where a proper query definition is crucial for the next steps. It is important to take into account that there are some weaknesses to overcome. Expert-based methods might become expensive when face-to-face meetings are held. In addition, experts may inherently carry personal or organizational biases, while computerized techniques show more objectivity [55]. However, to obtain a reliable result it is necessary to have proper software, with advanced data mining capabilities to process thousands of documents from myriad formats, as scientific documents and patents are not always consistent in formatting. This is expensive, requires a manual cleaning process and effectiveness depends greatly on the results retrieved from the search query. Thus, assuring the query reliability through expert validation is crucial to properly execute the CTI approach, which is to generate early warnings and aid in decision making.

The model applied to 3D bioprinting successfully generated insights that revealed the evolution of the field according to metrics such as publication and patent behavior, most prolific nations, top institutions. Additionally, it uncovered the main areas of research. It was found that both publication and patent rates exhibited exponential growth between 2010 and 2015, with an average annual growth of 20% and 40%, respectively. Exponential regression indicates that if the current trends continue, by 2020 there will be 2.4 times more articles and conference proceedings, and 5 times more patents per year than in 2015. It was also revealed that the USA and China are currently leading in the total number of scientific publications and patents in the field, and that there is significant evidence that this trend will continue. The patent analysis, which took into account the number of patents in different stages (applications, granted and inactive), revealed that most PFs belonging to top assignees are recent (filed in the last 5 years), and have not yet passed the application stage. This information highlights the key areas for the most recent innovations in the field.

One of the most important results was the PF knowledge clusters generated through the CTI methodology, and the identification of innovation drivers as defined by the experts. They revealed the strongest trends in current 3D bioprinting research. Tissue and organ engineering, which refers to the repair, maintenance or replacement of tissue, ranks in the top two positions in the knowledge clusters, and was also identified by the experts as one of the main innovation drivers. Bioinks are also currently being patented at a high rate. These are biological, biocompatible and bioabsorbable materials, such as polylactic acid and polyethylene glycol, among others, that can be printed while maintaining their integrity and structure, and keeping cells alive. New systems for 3D bioprinting are being investigated, as well. Vascularization is another of the biggest fields of innovation at present. This refers to the development of new ways to repair and maintain vascularization (development of blood vessels) of tissues or organs. Moreover, 3D bioprinting is also being used to create anatomical and pharmaceutical models, and is being used in regenerative medicine as well.

3D bioprinting has great implications for policy makers. 3D printing is an emerging and promising technology [12] that will enable disruptive innovation in tissue engineering [56]. It holds tremendous potential for the health industry, especially to address organ donation demand, and for prevention and regenerative medicine. Future research strategies should focus on creating more complex tissue or organs, and implement robotic systems to boost process productivity to an industrial level [57].

3D bioprinting represents an innovative method for addressing major problems in health. Early adopters of this technology will have major opportunities in its subsequent use. However, it is necessary to bear in mind that important challenges are associated with 3D bioprinting. In light of our results that identify the current state of the field, future studies should identify technological constraints, regulations, standardizations, intellectual property rights and ethical issues surrounding 3D bioprinting.

Finally, we have shown how this CTI methodology can be applied to study emerging technological innovations, such as 3D bioprinting. This case study is just a single example of how this methodology can be used to assess ongoing research trends and expose the knowledge landscape. The scope of the methodology is vast, and we expect that it can be successfully implemented to better understand any emerging technologies that are currently coming onto the market.

## Supporting information

S1 Appendix Search queries. (DOCX)

S1 File Scopus data import. (ZIP)

S2 File WoS data import. (ZIP)

S3 File Patent data import. (ZIP)
