# Supplementary material for: Scientometric and patentometric analyses to determine the knowledge landscape in innovative technologies: The case of 3D bioprinting
Source: PLoS One. 2017 Jun 29;12(6):e0180375. doi: 10.1371/journal.pone.0180375 (PMC5491216; doi:10.1371/journal.pone.0180375)
Supplement: S1 Appendix — (DOCX) [file pone.0180375.s001.docx]

**S1 Appendix. Search queries.**

Global query:

(((bio-fabricat* OR biofabricat* OR bio fabricat* OR bioprint* OR bio print* OR bio-print* OR bioink OR bio-ink OR “bio ink”)OR (((three dimensional OR 3d OR 3-d OR 3 d OR three-d or three d OR additive OR freeform OR desktop) wd1 (print* OR manufactur* OR fabricat*) OR rapid prototyp* OR layer by layer OR layer-by-layer )AND(((cell OR bone OR tissue OR organ OR bioscaffold* OR bio scaffold OR bio-scaffold OR biomimetic* OR skin OR cartilage OR scaffold*) wd1 (print*)) OR(tissue engineer* OR regenerative medicine OR biomedic* OR cancer model* OR biomanufact*)))) AND NOT ((stereoscopic* OR oxidation product OR streaming interactive OR nanoweb or nano web OR nano-web OR non halogen OR non-halogen OR ((food* OR feed* OR liquid*) w2 additive*) OR media access control OR multi-wafer 3D CAM cell OR 3-sigma or three sigma or rheolog* additive* OR vibration isolator* OR toilet OR paper OR transistor* OR light emitting OR diode OR solar cell OR spectophotom* OR spectroscop*)))

Scopus query:

((((TITLE-ABS-KEY((cell OR bone OR cartilage OR tissue OR organ OR scaffold* OR bioscaffold* OR "bio scaffold" OR bio-scaffold OR biomimetic* OR skin ) PRE/1 (print*))) OR (TITLE-ABS-KEY((tissue PRE/0 engineer*) OR "regenerative medicine" OR biomedic* OR (cancer PRE/0 model*) OR biomanufactur*))) AND (TITLE-ABS-KEY(((("three dimensional" OR 3d OR 3-d OR "3 d" OR three-d OR "three d" OR additive OR freeform OR desktop) PRE/1 (print* OR manufactur* OR fabricat*)) OR ((rapid PRE/0 prototyp*) OR "layer by layer" OR layer-by-layer))))) OR (TITLE-ABS-KEY(bio-fabricat* OR (bio PRE/0 fabricat*) OR biofabricat* OR bioprint* OR (bio PRE/0 print*) OR bio-print* OR bioink OR bio-ink OR "bio ink"))) AND NOT (TITLE-ABS-KEY (stereoscopic* OR "oxidation product" OR (streaming PRE/0 interactive) OR nanoweb OR "nano web" OR nano-web OR "non halogen" OR non-halogen OR ((food* OR feed* OR liquid* ) W/2 additive*) OR "media access control" OR "multi-wafer 3D CAM cell" OR 3-sigma OR "three sigma" OR "3 sigma" OR three-sigma OR (rheolog* PRE/1 additive*) OR (vibration PRE/1 isolator*) OR toilet OR paper OR transistor* OR "light emitting" OR diode OR "solar cell" OR spectophotom* OR spectroscop*)) AND (LIMIT-TO(PUBYEAR,2016) OR LIMIT-TO(PUBYEAR,2015) OR LIMIT-TO(PUBYEAR,2014) OR LIMIT-TO(PUBYEAR,2013) OR LIMIT-TO(PUBYEAR,2012) OR LIMIT-TO(PUBYEAR,2011) OR LIMIT-TO(PUBYEAR,2010) OR LIMIT-TO(PUBYEAR,2009) OR LIMIT-TO(PUBYEAR,2008) OR LIMIT-TO(PUBYEAR,2007) OR LIMIT-TO(PUBYEAR,2006) OR LIMIT-TO(PUBYEAR,2005) OR LIMIT-TO(PUBYEAR,2004) OR LIMIT-TO(PUBYEAR,2003) OR LIMIT-TO(PUBYEAR,2002) OR LIMIT-TO(PUBYEAR,2001) OR LIMIT-TO(PUBYEAR,2000)) AND (LIMIT-TO(DOCTYPE,"ar" ) OR LIMIT-TO(DOCTYPE,"cp"))

WoS query:

(((((TS=((((((((((cell) OR (bone)) OR (cartilage)) OR (tissue)) OR (organ)) OR (scaffold*)) OR (bio-scaffold)) OR (biomimetic*)) OR (skin)) NEAR/1 (print*))) OR (TS=(((((((tissue) (PRE) (0) (engineer*)) OR ("regenerative medicine")) OR (biomedic*)) OR ((cancer) (PRE) (0) (model*))) OR (hydrogel)) OR (biomanufact*)))) AND (TS=((((((((((("three dimensional") OR (3d)) OR (3-d)) OR ("3 d")) OR (three-d)) OR ("three d")) OR (additive)) OR (freeform)) OR (desotoi)) NEAR/1 (((print*) OR (manufactur*)) OR (fabricat*))) OR ((((rapid) NEAR/1 (prototyp*)) OR ("layer by layer")) OR (layer-by-layer))))) OR (TS=(((((bio-fabricat*) OR (biofabricat*)) OR (bioprint*)) OR (bio-print*)) OR (bio-ink)))) NOT (TS=((((((((((((((((((((stereoscopic*) OR ("oxidation product")) OR ("streaming interactive")) OR (nano-web)) OR (non-halogen)) OR ((((food*) OR (feed*)) OR (liquid*)) NEAR/2 (additive*))) OR ("media access control")) OR ("multi-wafer 3D CAM cell")) OR (3-sigma)) OR (three-sigma)) OR ((rheolog*) NEAR/1 (additive*))) OR ((vibration) NEAR/1 (isolator*))) OR (toilet)) OR (paper)) OR (transistor*)) OR ("light emitting")) OR (diode)) OR ("solar cell")) OR (spectophotom*)) OR (spectroscop*)))) AND (DT=((Article) OR ((Proceedings) (Paper))))

Patent query:

TAC: (((bio-fabricat* OR biofabricat* OR bioprint* OR bio-print* OR bioink OR bio-ink) OR (((three-dimensional OR 3d OR 3-d OR three-d OR additive OR freeform OR desktop) wd1 (print* OR manufactur* OR fabricat*) OR rapid prototyp* OR layer-by-layer) AND (((cell OR bone OR tissue OR organ OR bioscaffold* OR bio-scaffold OR biomimetic* OR bio-mimetic* OR skin OR cartilage OR scaffold*) wd1 (print*)) OR (tissue engineer* OR regenerative medicine OR biomedic* OR cancer model* OR biomanufactur*))))) NOT (TAC:(stereoscopic* OR oxidation product* OR streaming interactive OR nanoweb OR nano-web OR nonhalogen OR non-halogen OR ((food* OR feed* OR liquid*) w2 additive*) OR media access control OR multi-wafer 3D CAM cell OR 3-sigma OR three sigma OR rheolog* additive* OR vibration isolator* OR toilet OR paper OR transistor* OR light emitting OR diode OR solar cell OR spectophotom* OR spectroscop*)) AND PBD: [2000 to 2016-06-24]
