# Supplementary figures and images for: Scientometric and patentometric analyses to determine the knowledge landscape in innovative technologies: The case of 3D bioprinting
Source: PLoS One. 2017 Jun 29;12(6):e0180375. doi: 10.1371/journal.pone.0180375 (PMC5491216; doi:10.1371/journal.pone.0180375)

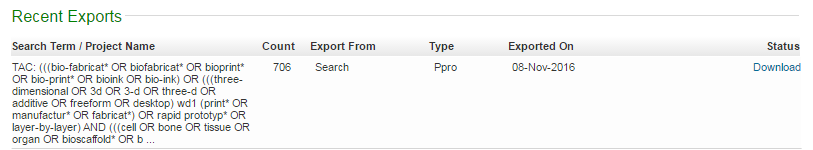

Supplement: S3 File — (ZIP) [file pone.0180375.s004.zip › Patseer Export of original patents (before cleaning).PNG]
